# Coinfection of Leprosy and American Cutaneous Leishmaniasis: A Case Report From Ceará, Brazil

**DOI:** 10.1155/carm/3062648

**Published:** 2026-09-06

**Authors:** Moacir Pereira Leite Neto, Jordannia Oliveira Fernandes, Ana Carolina Carvalho Ferraz, Julia Graziele Alves Marelli, Marcos Antônio Pereira de Lima, Cláudio Gleidiston Lima da Silva, Ligia Regina Franco Sansigolo Kerr, Ricardo Queiroz Gurgel

**Affiliations:** ^1^ Faculty of Medicine, Federal University of Cariri, Barbalha, Ceará, Brazil; ^2^ Postgraduate Program in Health Sciences and Parasitic Biology at the Federal University of Sergipe, Aracaju, Sergipe, Brazil; ^3^ Faculty of Medicine, Regional University of Cariri, Barbalha, Ceará, Brazil, urca.br; ^4^ Department of Community Health, Federal University of Ceará, Barbalha, Ceará, Brazil, ufc.br

**Keywords:** case reports, coinfection, cutaneous, diagnosis, differential, leishmaniasis, leprosy, pathology, surgical

## Abstract

Leprosy and American cutaneous leishmaniasis are endemic diseases in Brazil that may coexist in the same host, particularly in coendemic regions. We report the case of a 68‐year‐old woman presenting with a six‐month history of an ulcerated lesion on the left knee and concomitant disseminated annular plaques associated with sensory loss, madarosis, and peripheral nerve thickening. Histopathological examination of the ulcer demonstrated findings compatible with cutaneous leishmaniasis; immunohistochemistry using CD1a highlighted structures compatible with amastigotes, acknowledging its limitations. Biopsy of the plaques revealed a chronic granulomatous dermatitis with numerous acid‐fast bacilli, corroborating multibacillary leprosy in the appropriate clinical context. The patient was treated with multidrug therapy for leprosy and intralesional meglumine antimoniate for leishmaniasis, with favorable outcomes. To the best of our knowledge, this is the first published report of such coinfection in Ceará. This case highlights the importance of sampling lesions with distinct morphologies and integrating clinical, histopathological, and laboratory findings in endemic settings.

## 1. Introduction

Leprosy is caused by *Mycobacterium leprae*, an acid‐fast bacillus, and clinical manifestations may include skin lesions such as patches, plaques, or nodules and peripheral nerve involvement [1]. The most widely used classification is that of Ridley–Jopling, which establishes the following forms: (1) Tuberculoid, which presents a low bacillary load, with negative bacilloscopy, and a Th1‐type immune response pattern, characterized by an intense cellular response, with macrophage activation and the formation of compact granulomas that limit bacterial proliferation, clinically represented by early asymmetrical neuropathy, with thickened nerves and loss of sensitivity in a few nerves, and few well‐defined skin lesions, in hypopigmented or erythematous plaques or macules, with raised and anesthetic borders; (2) Borderline tuberculoid, with a low to intermediate bacillary load, a predominantly Th1‐type immune response pattern, but with a growing Th2 component, clinically represented by asymmetrical neuropathy and multiple skin lesions of the plaque or annular type with well‐defined borders and a center with a normal or scar‐like appearance; (3) Borderline dimorphic, with intermediate to high bacillary load, immunology with an unstable balance between Th1 and Th2 responses, with Th2 predominance, and clinical expression of asymmetrical neuropathy, with moderate neuritis and multiple skin lesions of the plaque, annular type, or with poorly defined borders and foveolar appearance; (4) Borderline virchowian, with high bacillary load, predominantly Th2 immunological pattern, with a predominant humoral response and weak cellular response, favoring bacterial dissemination, symmetrical and extensive, with neuritis in multiple nerve trunks, and multiple skin lesions, which may be macules, papules, nodules, or plaques, reddish or rusty in color, with symmetrical distribution; and (5) Virchowiana, with a high bacillary load, strongly positive bacilloscopy, and the presence of “globi” (clusters of bacilli within macrophages), Th2/regulatory type immunological polarization, with a dominant humoral response and abundant antibody production, and a suppressed cellular response, allowing for significant bacillary proliferation. Clinically, it presents with polyneuropathy and numerous symmetrical skin lesions, evolving from macules to nodules and diffuse skin infiltration, potentially leading to leonine facies, as well as alopecia of the eyebrows and eyelashes [2, 3].

American cutaneous leishmaniasis (ACL) is caused by protozoa of the genus *Leishmania*, the main species in Brazil being *Leishmania amazonensis*, *Leishmania guyanensis*, and *Leishmania braziliensis* [1]. Transmission is via phlebotomine vectors, affecting the skin and mucous membranes [1, 4].

The most common clinical manifestations are painless, ulcerated skin lesions with a rounded or oval shape, well‐defined and raised edges, a reddish base, and coarse, single or multiple granulations, or destructive lesions located in the mucous membranes, generally of the upper airways [1].

Coinfection with *Mycobacterium leprae* and a protozoan of the genus *Leishmania* is rarely reported as a clinically evident simultaneous coinfection, but it is increasingly recognized in coendemic regions, and we identified publications from the last two decades with case reports in Northeast Brazil [5], São Paulo [6], and Southeast Brazil [7], a seroprevalence study in Nicaragua and Honduras in Central America [8], and also a retrospective cohort study from the national notification system databases identified 28,204 patients with leprosy and 24,771 patients with cutaneous leishmaniasis between 2008 and 2017, with 414 patients affected by both diseases during that period [9].

Leprosy and ACL are endemic pathologies in South America and share clinical and immunological host manifestations that depend on the competence of the cell‐mediated immune response. Thus, it is thought that epidemiological factors, such as living in endemic areas and being a population at risk, and intrinsic biological factors, demonstrated by a reduced Th1 cell response, may be the reason for coinfection [10].

In cases of tuberculoid leprosy, there is a Th1 immune response, with a well‐defined granuloma infiltrated by CD4+ T lymphocytes, epithelioid cells and multinucleated giant cells, production of IFN‐γ and IL‐2, and activation of macrophages, while in lepromatous leprosy, there is a Th2 response, characterized by the production of antibodies, production of IL‐4, IL‐5, and IL‐10, and negative regulation of cellular immunity caused by the Th1 response [11].

In clinically evident leishmaniasis, there is an initial innate immune response by macrophages and natural killer cells, and an adaptive Th1 immune response caused by CD4+ T cells that produce IFN‐γ, TNF‐α, and IL‐12, which can lead to limitations or spontaneous cure, and a Th2 response with the production of IL‐4, IL‐5, and IL‐10, leading to the activation of humoral responses that are ineffective against the intracellular parasite [12].

We report the case of a patient with an ulcerated lesion on the left knee and diffusely scattered annular skin lesions throughout the body. The relevance of this report lies in the scarcity of cases of coinfection with leprosy and ACL, highlighting the importance of early recognition of these conditions for appropriate clinical management. Leprosy requires prolonged polychemotherapy, while ACL requires the use of leishmanicidal agents, such as pentavalent antimonials [13]. Concern about possible drug interactions and the recipient’s response during treatment for both types of immunity is important.

## 2. Case Presentation

**FIGURE 1:**
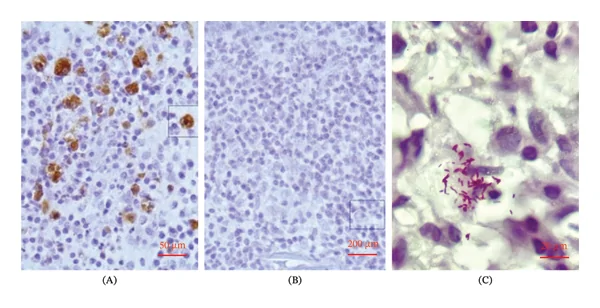
(A) Initial presentation at the first consultation (before biopsy and treatment), showing erythematous–squamous anesthetic plaques on the lower limbs (leprosy) and the ulcerated lesion on the left knee with a crusted base and well‐defined raised borders (ACL); (B) infiltrative plaque lesions on the forehead and madarosis of leprosy; and (C) follow‐up evaluation at the second consultation, approximately one month after the skin biopsy and prior to the start of treatment, showing the persistent anesthetic plaques and the evolution of the ulcerated lesion on the left knee, now presenting the biopsy site and initial healing changes.

A 68‐year‐old female patient with a history of systemic arterial hypertension, diabetes mellitus, and dyslipidemia was referred from a primary healthcare unit to a reference service for tropical diseases, where she was seen on August 9, 2024, due to an ulcerated lesion on her left knee that had been evolving for six months. During the clinical evaluation, in addition to the ulcerated lesion with a crusted base and well‐defined raised borders (Figure 1A), annular lesions were observed diffusely disseminated throughout the upper and lower limbs and abdomen. The latter presented as raised plaques with erythematous borders and a hypochromic center, associated with loss of tactile and thermal sensitivity (Figure 1C). Furthermore, the patient presented with madarosis, infiltrations in the nostrils and auricle (Figure 1B), as well as thickening of the ulnar, radial, and fibular nerves, and hypoesthesia in the territory of 26 peripheral nerves. Given the clinical picture, the diagnostic hypothesis of coinfection with leprosy and ACL was raised. Approximately one month after the skin biopsy, a follow‐up clinical reassessment was performed (Figure 1C), which documented the local evolution of the ulcer and the persistence of the anesthetic plaques.

**FIGURE 2:**
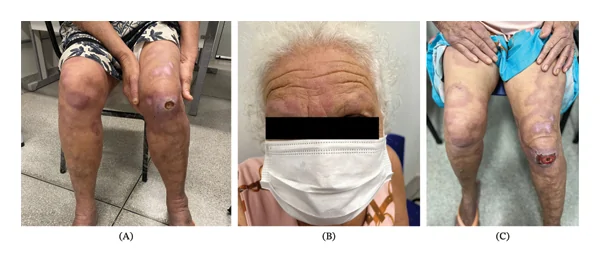
(A) Immunohistochemistry for CD1a (brown chromogen) highlighting structures compatible with leishmania amastigotes within phagocytic cells (original magnification × 400; scale bar = 50 μm). The highlighted squares represent leishmania amastigotes stained by immunohistochemistry with CD1a marker, with the nucleus and kinetoplast stained, filling the cytoplasm of the phagocytic cell. (B) Hematoxylin‐eosin staining of the same ulcerated lesion, showing no identifiable amastigotes (original magnification × 100; scale bar = 200 μm). (C) Fite–Faraco staining of the plaque biopsy, showing globi (clusters) of acid‐fast bacilli (red) within macrophages, characteristic of multibacillary leprosy (original magnification × 1000; scale bar = 20 μm).

To confirm the diagnosis, on the same day as the first evaluation, the patient underwent laboratory tests, including a complete blood count, erythrocyte sedimentation rate, urea, creatinine, sodium, potassium, calcium, magnesium, fasting blood glucose, aspartate aminotransferase, alanine aminotransferase, creatine phosphokinase, thyroid‐stimulating hormone, free T4, urinalysis, and stool parasitological examination, and all tests showed normal results. Furthermore, an acid‐fast bacilli test was performed on the lymphatic fluid, which was positive, and biopsies of the skin lesions were performed for histopathological examination and immunohistochemical studies. The biopsy of the ulcer on the left knee showed findings compatible with cutaneous leishmaniasis, and the immunohistochemistry for CD1a highlighted structures compatible with amastigotes (Figure 2A). The plaque biopsy showed chronic granulomatous lymphocytic dermatitis with numerous acid‐fast bacilli (Fite–Faraco staining), corroborating multibacillary leprosy in the clinical context, which was consistent with the positive bacilloscopy.

The histopathological examination of the biopsy tissue from the ulcerated lesion on the left knee showed findings consistent with cutaneous leishmaniasis, highlighting that immunohistochemistry for CD1a revealed structures consistent with amastigotes (Figure 2A), while hematoxylin‐eosin staining (Figure 2B) did not reveal identifiable amastigotes.

**FIGURE 3:**
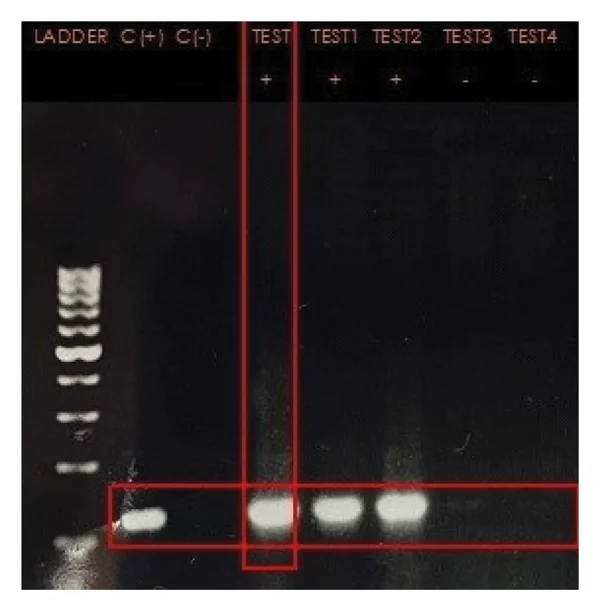
Conventional PCR for minicircular DNA from the kinetoplast of *Leishmania braziliensis* (2.5% agarose gel electrophoresis). M: molecular weight marker (ladder). C+: positive control. C–: negative control. Test: sample from the patient’s lesion (coinfected with leprosy and cutaneous leishmaniasis). TEST1 and TEST2: additional positive controls (two cases with confirmed cutaneous leishmaniasis). TEST3 and TEST4: negative controls (two cases without cutaneous leishmaniasis). An amplified band of expected size is observed in the test sample, confirming the presence of parasite DNA.

The DNA extracted from the biopsy was subjected to conventional polymerase chain reaction (PCR) assay for amplification of minicircular kinetoplast DNA fragments of *Leishmania braziliensis*, and the result showed the presence of parasite DNA in the lesion (Figure 3), confirming the leishmanial etiology, in agreement with the immunohistochemical findings.

The histopathological examination of the biopsy tissue from the annular plaque lesions showed chronic granulomatous lymphocytic dermatitis with numerous acid‐fast bacilli in Fite–Faraco staining (Figure 2C), corroborating multibacillary leprosy in the clinical context, which was consistent with the positive bacilloscopy.

The patient immediately began treatment for multibacillary leprosy with rifampicin, dapsone, and clofazimine for one year. One month after the biopsies were performed, topical treatment for acute leishmaniasis (ALL) with meglumine was initiated via intralesional infiltration directly at the base and edges of the lesion, once a week, for a total of 6 applications. The antiseptic effect was adjuvanted with a diluted 0.01% potassium permanganate solution, applied in moist compresses for 10 min, once a day, for 6 weeks. The response to treatment was effective; the leprosy spots disappeared, and the leishmaniasis ulcer healed. The neural thickening resolved, but the patient remained with mild sequelae of sensory deficit in the lower limbs.

## 3. Discussion

The current clinical case illustrates an unusual presentation of coinfection with leprosy and ACL, with manifestations that may overlap or even alter the clinical course of the other.

The diagnosis of leishmaniasis is based on criteria that combine epidemiological data, clinical characteristics, and laboratory test results, including the direct microscopy of tissue samples with Giemsa staining, immunohistochemistry using immunoperoxidase to detect amastigotes or *Leishmania* antigens, detection of *Leishmania* DNA by PCR, immunological hypersensitivity testing for *Leishmania* (Montenegro test), and serological tests using immunofluorescence and ELISA [14].

In the case presented, the single ulcerated lesion on the knee, with its classic characteristics of a crusted base and raised borders, was highly suggestive of leishmaniasis, and the CD1a antigen has been used because due to the commercial unavailability of immunohistochemical markers for specific *Leishmania sp*. antigens [15], the CD1a antigen has been used as an auxiliary method in the diagnosis of leishmaniasis [15–17], which is present in the plasma membrane of dendritic cells and thymocytes, and it is believed that amastigotes acquire this CD1a surface antigen after phagocytosis by dendritic cells, exposing CD1a on their own surface [15]; however, it is important to emphasize that its use has limitations such as false positives reflecting specificity problems, variability in sensitivity depending on the anti‐CD1a antibody clone used, and low sensitivity in samples with low parasite load and in the American continent [15, 17].

The disseminated annular lesions with erythematous borders and hypochromic center, associated with thickened nerve trunks, hypoesthesia in multiple territories, madarosis, and infiltration of the auricles allowed for the diagnosis of multibacillary leprosy (lepromatous leprosy) [18].

Skin ulcers are a complication of neuropathy in Hansen’s disease [19] but are usually with plantar localization [20]. In the patient’s case, the skin ulcer was located on the knee, and not in the plantar areas, and was not indicative of a leprosy lesion.

In the present case, the patient lives in a region endemic for both diseases, and the overlap of signs and symptoms required a detailed histopathological study of the two lesions since the dermatological presentations of these diseases can be atypical.

Annular lesions with hypoesthesia and neural thickening are virtually pathognomonic for leprosy. Regarding the single chronic ulcer with raised borders in an exposed area, it is also highly suggestive of cutaneous leishmaniasis, and a differential diagnosis should be established with bacterial ulcer, which, however, is painful and rapidly growing, while in leishmaniasis, it is generally painless, sporotrichosis can present as a chronic ulcer, but it is usually more verrucous and does not typically have such well‐defined and raised borders, and infection by atypical mycobacteriosis is also a possibility [1], but the epidemiology of the patient in this case does not support this possibility.

The diagnosis of leishmaniasis was corroborated by histopathological and immunohistochemical findings of the knee ulcer. The immunohistochemical study showed positivity for the CD1a marker; notably, the antibody outlined the membranes of intracellular amastigotes within phagocytic cells, thereby providing an auxiliary confirmation of leishmaniasis [21]. Concurrently, to address the systemic presentation, a separate biopsy of the plaque lesions revealed a classic granulomatous infiltrate filled with acid‐fast bacilli, confirming a coinfection with multibacillary leprosy.

The treatment was successful in addressing both infections concurrently but independently, as standard multidrug therapy for multibacillary leprosy includes rifampicin, dapsone, and clofazimine, while the treatment of leishmaniasis was conducted with intralesional infiltrations of meglumine antimoniate. It is important to highlight that a failure to diagnose cutaneous leishmaniasis would result in the absence of its treatment, which could lead to chronicity and expansion of the ulcer, secondary bacterial infection, mucocutaneous dissemination, misdiagnosis of the knee ulcer as an atypical leprosy reaction, and persistent pain and discomfort.

Coinfection with *Mycobacterium leprae* and a protozoan of the *Leishmania* can occur especially in endemic regions for both diseases, involving socioeconomic and environmental factors (epidemiological coincidence), but also modulation of the immune response, as both intracellular pathogens trigger an effective cellular immune response by Th1 lymphocytes to control the infection, and infection by one can modulate the levels of the immune response to the other [7]. The interaction of IFN‐γ and TNF‐α is related to the elimination of *Leishmania* and mycobacteria by macrophages [22]. A reduction in IFN‐γ and IL‐12 and an increase in IL‐10 and TGF‐β are observed in coinfected individuals, resulting in an immunosuppressive response and reducing the elimination capacity of *Mycobacterium leprae* and *Leishmania* [23]. If ACL is not treated, active *Leishmania* infection could act in a cross‐modulated immune response, producing cytokines such as IL‐10 and TGF‐β, which could intensify leprosy reactions.

This report contributes to expanding knowledge about coinfections in endemic areas, helping to improve diagnostic and management strategies for these diseases. Furthermore, this case report draws attention to the importance of further studies to be carried out to better understand the interaction between *Mycobacterium leprae* and *Leishmania sp*. and its clinical, immunological, and therapeutic implications.

## Author Contributions

Author contributions according to the Contributor Roles Taxonomy (CRediT): Moacir Pereira Leite Neto: conceptualization (conceived the project, defining the study objectives and methods), investigation (provided medical care to the patient, conducted literature review, and participated in case discussion), and writing–review and editing (participated in the review and final editing of the text and submitted the article to the journal); Jordannia Oliveira Fernandes: writing–original draft (participated in the literature review); Ana Carolina Carvalho: writing–original draft (drafted the project proposal submitted to the research ethics committee); Julia Graziele Alves Marelli: investigation (performed polymerase chain reaction procedures and participated in case discussion) and writing–review and editing (participated in the review of the article); Marcos Antônio Pereira de Lima: investigation (performed polymerase chain reaction procedures and participated in case discussion) and writing–review and editing (participated in the review of the article); Cláudio Gleidiston Lima da Silva: investigation (performed the anatomopathological examination) and supervision (provided guidance external to the main team); Ligia Regina Franco Sansigolo Kerr: writing–review and editing (participated in the review and translation of the article); Ricardo Queiroz Gurgel: supervision (provided mentorship external to the main team and led the planning of research activities) and writing–review and editing (participated in the review of the article).

## Funding

No funding was received for this manuscript.

## Disclosure

All authors have read and approved the final version of the manuscript.

## Consent

The patient participating in the research authorized through an informed consent form for the publication of the case report in a scientific journal and also authorized the use of images in the same publication, signed by her guardian (her son), after verbal explanations about the investigation, its reasons and importance, and the potential risks of the research. The research project for this case report was submitted to the Research Ethics Committee of the School of Medicine of the Federal University of Cariri for review, approved by opinion no. 7,256,191, issued on November 28, 2024. Authorization was also obtained from the dean of the School of Medicine of the Federal University of Cariri.

## Conflicts of Interest

The authors declare no conflicts of interest.

## Supporting Information

Additional supporting information can be found online in the Supporting Information section.

## Supporting information


**Supporting Information** CARE Checklist Case Coinfection.

## Data Availability

The clinical and histopathological data from the case report used to support the results of this study are restricted by the Research Ethics Committee of the School of Medicine of the Federal University of Cariri in order to protect patient privacy. The data are available to researchers who meet the criteria for access to confidential data at the School of Medicine of the Federal University of Cariri, telephone +558832219200, email famed@ufca.edu.br, and under the responsibility of Prof. Dr. Claudio Gleidiston Lima da Silva, email claudio.gleidiston@ufca.edu.br.
