# Supplementary material for: Coinfection of Leprosy and American Cutaneous Leishmaniasis: A Case Report From Ceará, Brazil
Source: Case Rep Med. 2026 Sep 6;2026:3062648. doi: 10.1155/carm/3062648 (PMC13547950; doi:10.1155/carm/3062648)
Supplement: Supplementary file 1 [file CARM-2026-3062648-s001.pdf]

CARE Checklist – Case Report

Manuscript title: Coinfection of Leprosy and American Cutaneous Leishmaniasis: A Case Report from Ceará, Brazil

Topic	CARE Item	Description	Addressed in Manuscript
Title	1	The words “case report” and the main phenomenon of interest appear in the title.	Yes – included in title.
Keywords	2	2–5 key words identifying diagnoses or interventions.	Yes – keywords provided.
Abstract	3a	Introduction – What is unique about this case?	Yes – rarity of coinfection highlighted.
Abstract	3b	Main symptoms and clinical findings.	Yes – ulcerated lesion, annular plaques, neuropathy.
Abstract	3c	Main diagnoses, interventions, and outcomes.	Yes – leprosy and ACL diagnoses; MDT and meglumine treatment.
Abstract	3d	Conclusion and lessons learned.	Yes – importance of sampling lesions and integrated diagnosis.
Introduction	4	Background and rationale for the case report.	Yes – epidemiology and immunology discussed.
Patient Information	5a	Demographic information.	Yes – 68-year-old female patient.
Patient Information	5b	Main symptoms and concerns.	Yes – chronic knee ulcer and disseminated plaques.
Patient Information	5c	Medical, family, and psychosocial history.	Partial – comorbidities described; family/social history not detailed.
Clinical Findings	6	Relevant physical examination findings.	Yes – madarosis, nerve thickening, hypoesthesia, plaques.
Timeline	7	Timeline of events.	Chronology described narratively.
Diagnostic Assessment	8a	Diagnostic methods used.	Yes – histopathology, immunohistochemistry, bacilloscopy, PCR.
Diagnostic Assessment	8b	Diagnostic challenges.	Yes – overlapping manifestations and limitations of CD1a discussed.
Diagnostic Assessment	8c	Diagnostic reasoning and differential diagnosis.	Yes – differential diagnoses discussed in discussion section.
Diagnostic Assessment	8d	Prognostic characteristics.	Not applicable.
Therapeutic Intervention	9a	Types of intervention.	Yes – multidrug therapy and intralesional meglumine.
Therapeutic Intervention	9b	Administration details.	Yes – weekly infiltrations and duration reported.
Therapeutic Intervention	9c	Changes in intervention with rationale.	No major changes reported.
Follow-up and Outcomes	10a	Clinician and patient-assessed outcomes.	Yes – healing of ulcer and regression of lesions.
Follow-up and Outcomes	10b	Important follow-up test results.	Yes – clinical resolution and residual sensory deficit described.
Follow-up and Outcomes	10c	Intervention adherence and tolerability.	Partial – treatment success described, adherence not specifically detailed.
Follow-up and Outcomes	10d	Adverse and unanticipated events.	No adverse events reported.
Discussion	11a	Strengths and limitations of management.	Yes – limitations of CD1a and rarity of case discussed.
Discussion	11b	Relevant medical literature.	Yes – comprehensive literature review included.

Discussion	11c	Scientific rationale and conclusions.	Yes – immunological interaction and diagnostic implications discussed.
Discussion	11d	Take-away lessons.	Yes – importance of recognizing coinfections in endemic areas.
Patient Perspective	12	Patient perspective or experience.	Not included.
Informed Consent	13	Patient informed consent obtained.	Yes – explicitly stated in manuscript.

General assessment: The manuscript substantially complies with CARE reporting guidelines.
